# Controversies in the Management of Lateral Pelvic Lymph Nodes in Patients With Advanced Rectal Cancer: East or West?

**DOI:** 10.3389/fsurg.2019.00079

**Published:** 2020-01-17

**Authors:** Jaime Otero de Pablos, Julio Mayol

**Affiliations:** Department of Surgery, Hospital Clínico San Carlos, Instituto de Investigación Sanitaria, Universidad Complutense de Madrid, Madrid, Spain

**Keywords:** locally advanced rectal cancer, lateral pelvic lymph node, lateral pelvic lymph node dissection, East vs. West, surgical oncology

## Abstract

The presence of lateral pelvic lymph nodes (LPLN) in advanced rectal cancer entails challenges with ongoing debate regarding the role of prophylactic dissection vs. neoadjuvant radiation treatment. This article highlights the most recent data of both approaches: bilateral LPLN dissection in every patient with low rectal cancer (Rb) as per the Japanese guidelines, vs. the developing approach of neoadjuvant radiotherapy as per Eastern countries. In addition, we also accentuate the importance of a combined approach published by Sammour et al. where a simple “one-size-fits-all” strategy should be abandoned. Rectal cancer treatment is well-established in Western countries. Patients with advanced rectal cancer will undergo radiation ± chemo neoadjuvant therapy followed by TME. In the Dutch TME trial, TME plus radiotherapy showed that the presacral area was the most frequent site of recurrence and not the lateral pelvic wall. Supporting this data, the Swedish study also concluded that LPLN metastasis is not an important cause of local recurrence in patients with low rectal cancer. Therefore, Western approach is CRM-orientated and prophylactic LPLN dissection is not performed routinely as the NCCN guideline does not recommend its surgical removal unless metastases are clinically suspicious. The paradigm in Eastern countries differs somewhat. The Korean study demonstrated that adjuvant radiotherapy without lateral lymph node dissection was not enough to control local recurrence and LPLN metastases. The Japanese Trial JCOG 0212 demonstrated the effects of LPLN dissection in reducing local recurrence in the lateral pelvic compartment. We agree with Sammour and Chang on the fact that rather than a mutual exclusivity approach, we should claim for an approach where all available modalities are considered and used to optimize treatment outcomes, classifying patients into 3 categories of LPLN: low risk cT1/T2/earlyT3 (and Ra) with clinically negative LPLN on MRI; Moderate risk (cT3+/T4 with negative LPLN on MRI) and high risk (clinically abnormal LPLN on MRI). Treatment modality should be based on detailed pretreatment workup and an individualized approach that considers all options to optimize the treatment of patients with rectal cancer in the West or the East.

## Background

The modern treatment of locally advanced rectal cancer (LARC), that is, stage II (T3-T4 and node negative) or stage III (node positive) disease according to the American Joint Committee on Cancer (AJCC) Staging Classification system, is based on the combination of multiple therapies, requiring a multidisciplinary approach. Surgery continues to play a major role in reducing local recurrence rates given the new developments in surgical technique, in addition to the improvements in neoadjuvant strategies and immunological therapy.

In patients with non-metastatic disease, achieving complete en-bloc resection of the rectum and any involved organ containing the primary tumor is the most significant predictive factor related to improved survival, being the results comparable to those in patients whose cancers did not invade adjacent structures (1). To ensure optimal outcomes, accurate preoperative assessment and patient selection are crucial.

Given the complex anatomy of the pelvis, the potential involvement of lateral pelvic lymph nodes (LPLN) in LARC represents a major challenge, and there is an ongoing debate regarding the role of prophylactic dissection compared to neoadjuvant chemoradiation to improve outcomes. Neoadjuvant chemoradiation therapy (CRT) followed by total mesorectal excision (TME) is the current standard of care for LARC in North America and Europe (2). Hesitation to perform lateral pelvic lymph node dissection might be based on a presumed increased morbidity. However, studies from Eastern countries, such as Japan, have reported an incidence of lateral lymph node involvement outside the field of TME from 10% to up to 25% in some cases of LARC, where no previous lymph nodes were identified (3–5).

In fact, some studies have recently suggested that chemoradiotherapy plus TME without lateral lymph node dissection (LLND) may not be adequate in patients where enlarged (greater than 7 mm) lymph nodes have been identified preoperatively to reduce local recurrence (6, 7).

Obviously, the optimal strategy to deal with LPLN in patients with locally advanced rectal cancer remains controversial. In this article, the most recent evidence for both approaches, neoadjuvant chemoradiotherapy proposed by European and North American guidelines, and bilateral pelvic lymph node dissection proposed by the Japanese guidelines, is reviewed.

## Importance of Lateral Lymph Node Metastasis

The lower rectum will drain following two major routes, one concur into the superior rectal artery and the inferior mesenteric artery and then into the para-aortic nodes, and the other leads into the middle and inferior rectal artery into the obturator and the terminal internal iliac nodes and external iliac nodes. As described for the standard TME approach, the vast majority of the lymph nodes from the first group will be systematically resected, whereas the second group of nodes will not be routinely resected according to the European and North American recommendations, unless they were recognized as metastatic.

**Figure 1:**
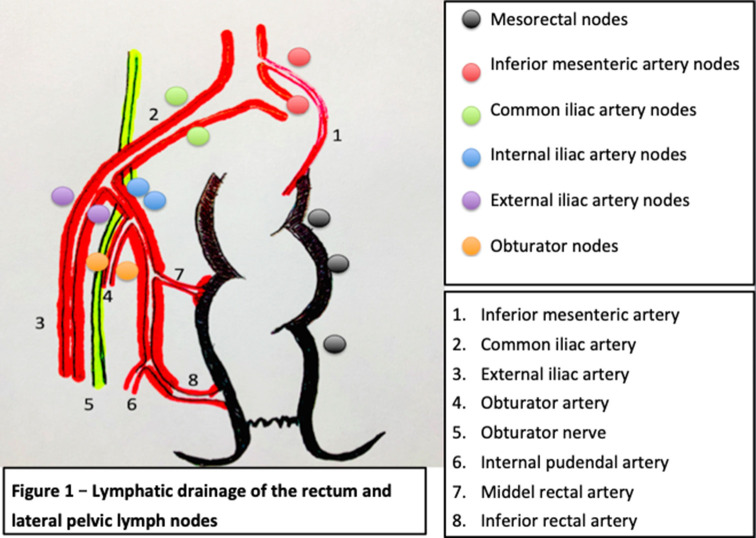
Lymphatic drainage of the rectum and lateral pelvic lymph nodes [modified and adapted from (9, 10)].

According to the TNM staging of the AJCC American Joint Committee on Cancer, the presence of pathological lymph nodes in the internal iliac group is considered as regional disease while lymph nodes in the external and common iliac nodes are treated as metastatic disease. In contrast, as per the Japanese guidelines, all of the lateral lymph nodes are considered regional nodes (8). The lymphatic drainage of the rectum is described in Figure 1. Cannessa et al. published on their anatomic study the amount of lymph nodes retrieved and their location in the hemipelvis from cadavers. They classified the nodes into three regions: presacral, hypogastric, and obturator group. This study showed that the highest incidence of nodal involvement was found along the internal pudendal artery region, the internal iliac artery region and the obturator region, considering these regions combined as a “*vulnerable field”* (11). The lymph nodes from the internal iliac artery were found predominantly above the pelvic nerve plexus but reaching the deep pelvic veins, hence, demanding a deep pelvic dissection of the neurovascular structures (11).

In addition, these three areas previously mentioned are relevant oncologically due to their closeness to the circumferential margin. It is believed that disease in this area is because of the lymphatic drainage from the lower rectum exceeds the mesorectum through the lateral ligament and then along the internal iliac artery and consequently into the obturator space. This theory explains why the obturator region has the highest rate of nodal involvement and it should be examined as a significant area of cancer spread in these tumors (11).

LPLNs have been proposed to be the major cause of local recurrence after curative resection in low rectal cancer, given that about 50% of the local recurrences occur in the lateral lymph node area with no evidence of distant metastasis (12).

Prognostic factors in patients with rectal cancer have been published in the literature including the level of the distal tumor edge, annularity, depth of invasion, number of metastatic nodes apart from LPLN, the presence of malignant nodes at the site of the superior rectal artery, preoperative CEA levels and histologic differentiation of the tumor (13).

## Prediction of Lateral Pelvic Lymph Node Metastasis

The incidence of lateral wall pelvic lymph node metastasis in locally advanced rectal cancer from all major studies published recently is summarized in Table 1, fluctuating from 7 to 24%. Presence of lymph nodes in the lateral pelvic compartment has a poor prognostic factor, with a 5-year survival rate of 25.1% compared to 74.3% in those with uninvolved nodes (26). Although locally advanced low rectal cancer treatment remains unclear, identification of risks factors for LPLN metastasis is crucial to select patients who may benefit from lateral pelvic lymph node dissection and/or neoadjuvant chemoradiotherapy.

**Table 1:** Incidence of lateral wall pelvic lymph node metastasis in locally advanced rectal cancer.

**References**	**Number of cases**	**Incidence LPLN metastasis**
Kinugasa et al. (14)	944	22%
Takahashi et al. (15)	764	8.60%
Fujita et al. (16)	204	11.90%
Ueno et al. (17)	244	17.30%
Min et al. (5)	151	23.80%
Fujita et al. (18)	351	7%
Matsumoto (19)	387	15.50%
Quadros et al. (20)	102	17%
Ogawa et al. (21)	222	20.30%
Ogawa et al. (21)	230	17%
Sugihara et al. (22)	930	13.80%
Kobayashi et al. (23)	784	14.90%
Fujita et al. (24)	210	22.40%
Akiyoshi et al. (25)	5,789	11.30%

Multivariate analysis from the Sugihara et al. study (22) demonstrated that female gender, moderately or poorly differentiated adenocarcinoma, low rectal tumor (as the distance from the anal verge decreases, the incidence of lateral pelvic nodes increases), tumor size equal or greater than 4 cm and stage T3-T4 rectal tumors were significantly associated with an increased rate of lateral pelvic lymph node metastasis, showing low rectal cancer and T3-T4 stage cancer the higher hazard ratios. Multiple studies (3, 13) have been published supporting these factors as well as some other studies such as Tan et al. (27) where positive lymphatic invasion was also identified as a risk factor. Besides, identification of metastatic lymph nodes in the pelvis on diagnostic imaging is a direct finding and may play an important role as well.

## Detection of Lateral Pelvic Lymph Nodes

The presence of metastatic lymph nodes in lower rectal cancer is essential as it may determine the patient's treatment. Nowadays, patients are provided with a wide range of imaging techniques along with ultrasound, CT pelvis, positron emission tomography-CT and MRI. MRI is considered highly accurate in detecting lateral pelvic nodes, with a 67% sensitivity, 75% specificity, and 73% overall accuracy (28). Some groups such as LOREC (Low Rectal Cancer Study Group) or the Japanese Society for Cancer of the Colon and Rectum, suggesting nodal size cutoff (>5, >7, >8 mm on the long or short axis) measured on MRI and/or CT scan in order to determine which nodes might be considered as pathological. Also, not only the size, but the nodal margins and nodal characteristics may suggest more reliability as indicators of malignancy (29).

## Evidence of Neoadjuvant Treatment: Management in North America and Europe

There are two distinct hypotheses to explain local recurrence in patients with rectal cancer. While surgeons in Eastern countries blame LPLN as the main cause of local recurrence in these patients who have been subjected to total mesorectal excision (TME), surgeons from Europe and North America have concentrated on surgical clearance of the circumferential resection margin (CRM) (30).

Although many terms have been proposed to define circumferential resection margin, it is widely accepted as the “closest distance between the radial resection margin and the tumor tissue by either direct tumor spread, areas of neural or vascular invasion, or the nearest involved lymph node” (31). Many studies have demonstrated the importance of CRM as an independent prognostic factor of local recurrence and long term survival (32, 33), including the first study published regarding this topic by Quirke et al. (34). The optimal cutoff for defining positive CRM is debatable. Most of the literature, including the NCCN guidelines, defines a positive CRM as tumor within 1 mm of the cut surface (35).

Rectal cancer treatment is well-established in North America and Europe. In patients with clinically early disease (T1-T2, N0), TME alone is sufficient (35) as these cancers are less likely to have spread to regional lymph nodes. However, patients with locally advanced rectal cancer usually undergo radiation ± chemo neoadjuvant therapy followed by TME to minimize locoregional recurrence rate and to improve long-term cancer-specific outcomes (35).

The Dutch Rectal Cancer Trial was the first to address the beneficial effects of preoperative radiotherapy plus TME in reducing local recurrence rates in stage II and stage III rectal cancers from 11% in the non-preoperative radiotherapy group to 5% in the preoperative radiotherapy group after 10-year (*P* < 0.0001) as well as it demonstrated that the presacral area, and not the lateral pelvic wall, was the most frequent site of recurrence (36). Supporting these data, the Swedish study (37) also concluded that metastasis to the LPLN is not an important cause of local recurrence in patients with low rectal cancer.

Neoadjuvant chemoradiation therapy has proved to be advantageous in ensuring better surgical clearance of the central pelvis as well as in securing the circumferential margin. With this treatment modality, the entire pelvis, including lymph nodes from the central and lateral compartments are covered, hence, minimizing the potential risk of cancer leakage. European and North American associations have strongly supported the notion that the circumferential resection margin is one of the most important prognostic factors related to local control in locally advanced rectal cancer surgery.

Many randomized trials have described the beneficial effects in local control of neoadjuvant therapy (38, 39). Nevertheless, when the Dutch TME trial results were compared with data from the National Cancer Center Hospital in Japan (40), the rates of local control were not different between TME preceded by radiotherapy vs. TME with lateral pelvic lymph node dissection. Even though, because of the vast variety of outcomes, the need for LPLN dissection in patients who have undergone preoperative chemoradiation remains unclear.

The role of radiotherapy for this therapeutic strategy has been broadly accepted as it reduces local recurrence when combined with surgical resection and enhances survival when used in multidisciplinary treatment. However, it is not used routinely in all rectal cancer because of the toxicity, associated complications and low local recurrence rates in stage I low rectal cancer.

Supporting the North American and European trend, two studies by Japanese groups have been published. Watanabe et al. (8), with a retrospective study, and Nagawa et al. (41), with a small scale randomized controlled trial comparing preoperative radiation with and without LPLN dissection, concluded that LPLN dissection is not necessary as a curative option for patients with advanced low rectal cancer, and that preoperative radiotherapy can be employed as an alternative to LPLN dissection, reducing the irreversible risks of postoperative functional disability.

The effects of pre or postoperative radiotherapy regimens have been evaluated in different randomized controlled trials (38, 39). As a consequence of the fractionation schedules and interval-to-surgery differences between the European and the American reports, a direct correlation between these two procedures using published literature would be very ambitious.

As Kim described in his systematic review about “*Controversial issues in radiotherapy for rectal cancer*” (42) “*preoperative radiotherapy has biological advantages as intact blood vessels and higher oxygenation status can contribute to higher radiosensitivity. The downsizing effect can be attributed to increased resectability and sphincter preservation rate*” (42). In contrast, Kim also mentions in his review that “*the benefits of postoperative RT include a better patient selection with high risk of local recurrence that can be verified by surgical and or pathological findings*” (42).

Therefore, North American and European societies recommend a CRM-orientated approach and prophylactic LPLN dissection is not indicated routinely. Only when clinically suspicious lateral pelvic lymph node metastasis are confirmed, surgical removal is indicated (35).

## Prophylactic Lymph Node Dissection: Management in Asia

Despite the known benefits of selective preoperative chemo-radiation prior to TME where literature consistently has shown lower locoregional recurrence rates, the long-term advantages of this approach has not been fully established yet (43).

Lateral pelvic lymph node dissection was first described in Western countries in 1950's, however, because of its significant morbidity and postoperative functional disabilities, was abandoned (44).

As Sammour et al. described in their article (45), in terms of anatomy and biology, it is more appropriate to consider lateral node involvement for a tumor in the mid and low rectum as locoregional, rather than as distant disease. In Japan, the evolution in the surgical oncology approach has been toward lymph node clearance and, as a result, lateral pelvic nodes have been considered local-regional disease from the outset. Hence, Eastern countries, especially Japan, has derived to add surgical therapy of this compartment as part of the standard of care for these patients with low rectal cancer.

According to the Japanese Society for Cancer of the Colon and Rectum (JSCCR) guidelines and based on the fact that 15–20% of patients with T3-T4 rectal cancer located below the peritoneal reflection have already metastasis in the lateral pelvic compartment, LPLN dissection is indicated when the lower edge of the tumor is located distal to the peritoneal reflection and when the tumor invades beyond the muscularis propria, or when there are enlarged lateral pelvic lymph nodes present on CT/MRI (8). Following these recommendations, LPLN dissection is expected to decrease the intrapelvic recurrence by 50% and improve the 5 year survival by 8–9% (8).

In 2017, the Colorectal Cancer Study Group of Japan Clinical Oncology Group published a randomized controlled non-inferiority trial designed to test the non-inferiority of the TME alone in stage II and III low rectal cancer with TME plus LPLN dissection in terms of efficacy (46). Some 701 patients were randomized and the non-inferiority of TME alone to TME with LPLN dissection could not be confirmed. Also, this study showed a decrease in local recurrence, especially in the lateral pelvis, supporting the need of TME plus LPLN dissection in locally advanced low rectal cancer (46).

Following the trend of an aggressive surgical approach as previously suggested by the JSCCR in 2008, Kim et al. (47) analyzed more than 350 patients who underwent preoperative chemoradiation without LPLN dissection and determined that lateral pelvic recurrence was a major type of locoregional recurrent disease, hence, suggesting this approach was not sufficient to ensure clearance of the lateral pelvis.

In addition, two leading units in Japan reported their experience with prophylactic LPLN dissection in 1,191 consecutive patients (48). They demonstrated an incidence of LPLN metastasis of 15.8 to 19.1% and showed a better control with lateral pelvic lymph node dissection. They published an overall survival of 45–53% among patients with LPLN metastasis and 81–81.7% among those patients with no LPLN metastasis.

On the other hand, prophylactic LPLN dissection has some drawbacks, which are longer operative time, higher intraoperative blood loss and higher rate of postoperative complications including urinary and sexual dysfunction as mentioned previously (41).

The polemic whether preoperative chemoradiation plus TME is superior or equivalent to TME plus LPLN dissection will not likely be elucidated soon as it will need further randomized controlled trials to compare both approaches.

Morbidity associated with LPLND has been a major concern (Table 2). Even though new modern techniques and minimally invasive surgery have reduced postoperative complications, it inevitably involves some risk of causing injury to vessels and nerves, hence compromising sexual and voiding functions. After the meta-analysis published by Petersen et al. (56), extended lymphadenectomy is not routinely performed anymore as it showed a 3.7 times higher risk or urinary dysfunction and a 2.08 times higher risk of urinary retention.

**Table 2:** Postoperative functional outcomes of cases with lateral pelvic lymph node dissection.

**References**	**Evaluated function**	**Functional result**
Sugihara et al. (49)	Sexual function	29,6% Did not maintain male sexual function
Matsuoka et al. (50)	Urinary function	86% Dysuria
		40% Urinary incontinence
		54% Change in bladder sensation
		25% Needed Intermitent Catherization
Maeda et al. (51)	Urinary function	15% Minor disturbance
	Sexual function	27% Partial or total impotence
		11% Retrograde ejaculation
Col et al. (52)	Urinary function	58% Urinary incontinence
		16% Urinary retention
Kyo et al. (53)	Urinary function	13,3% Difficulty in emptying the bladder
		13,3% Minor incontinence
	Sexual function	50% Decreased sexual activity
		50% Erectile dysfunction
		90% Ejaculatory dysfunction
Saito et al. (54)	Sexual function	79% Sexual dysfunction
Ito et al. (55)	Urinary function	59% Urinary dysfunction

## Suggested Treatment Modality

The paradigm in North America and countries from Europe differs from the Western approach. The Korean study (41) demonstrated that adjuvant radiotherapy without lateral lymph node dissection was not enough to control local recurrence and LPLN metastases. The Japanese Trial JCOG 0212 (46) showed that LPLN dissection reduced local recurrence in the lateral pelvic compartment.

Publications in Western countries such as the CAO/ARO/AIO-94 randomized trial by Sauer et al. and the randomized trial by Sauer et al. (38) and Sebag-Montefiore et al. (39) have proved that neoadjuvant therapy improves local control. However, articles such as Moriya's (57) published in Japan have proved adequate local control with LPLN dissection without neoadjuvant treatment. As it has been already published in the literature by van Gijn et al. (36) and Fujita et al. (46) the addition of chemoradiation with TME does not improve survival and is not sufficient for eradicating LPLN. Nonetheless, postoperative adjuvant chemotherapy using 5-Fluorouracil has offered an advancement in patient survival with resectable disease (56, 58).

Patients selection plays an important role when choosing which patients are going to receive beneficial effects from LPLN dissection. As mentioned previously, size and heterogeneity of the nodes are reliable in predicting malignancy of the nodes. Also, the response to chemoradiotherapy is a good prognostic indicator. Kim et al. (59) proved that non-suspicious LPLN group and LPLN that responded to chemoradiotherapy had no significant differences in the recurrence free survival and overall survival.

The article by Sammour and Chang (45) brought up appealing questions about the different paths North America-Europe and Asia, especially Japan, would manage LPLN.

In our opinion, the “one-size-fits-all” strategy should be abandoned. As Sammour and Chang proposed (45), it is reasonable to advocate that all available modalities have to be considered and used to optimize treatment outcomes. For this purpose, patients who are candidates for curative-intent treatment should be stratified depending on their risk to have LPLN metastasis (as shown in Table 3) in order to select the best option to manage the pelvic compartment. Until further evidence is available, patients would be better managed as follows:

- Low risk: TME alone would be sufficient.
- Moderate risk: treatment would consist of neoadjuvant treatment + TME or TME + LPLN dissection (to date, there is no clear consensus on which approach will fit best for these patients).
- High risk: these patients should undergo neoadjuvant treatment + TME + LPLN dissection (particularly if the abnormal nodes do not respond to neoadjuvant treatment).

**Table 3:** LPLN risk stratification of patients with low rectal cancer for lateral compartment management purpose according to Sammour and Chang.

**Risk**	**TNM**	**LPLN status on MRI**
Low	cT1, T2, early T3	Negative
Moderate	cT3+, T4	Negative (potential microscopic disease)
High	–	Abnormal (macroscopic disease)

Pursuing Sammour and Chang recommendations, treatment modality should be based on detailed pretreatment workup and an individualized approach that considers all options to optimize the treatment of patients with rectal cancer worldwide (44).

A future randomized clinical trials comparing TME + LPLN dissection vs. chemoradiotherapy + TME, and/or chemoradiotherapy + TME + LPLN dissection vs. chemoradiotherapy + TME may be needed to clarify the true benefit of prophylactic LPLN dissection for locally advanced low rectal cancer.

In summary, this review analyses the potential advantages and disadvantages of the two distinct approaches popular in North America and Europa, and Asia, for the treatment of advanced rectal cancer. The importance of preoperative tumor staging, the decision making process and the use of all therapeutic modalities for patients according to the risk of metastatic LPLNs are discussed, acknowledging the limitations of available evidence coming from both East and West.

## Author Contributions

JM and JO have made substantial contributions to conception and design, and/or acquisition of data, and/or analysis and interpretation of data, have participated in drafting the article or revising it critically for important intellectual content, and have given final approval of the version to be submitted.

### Conflict of Interest

The authors declare that the research was conducted in the absence of any commercial or financial relationships that could be construed as a potential conflict of interest.
