# Corrigendum: Controversies in the Management of Lateral Pelvic Lymph Nodes in Patients With Advanced Rectal Cancer: East or West?

**DOI:** 10.3389/fsurg.2020.00041

**Published:** 2020-07-22

**Authors:** Jaime Otero de Pablos, Julio Mayol

**Affiliations:** Department of Surgery, Hospital Clínico San Carlos, Instituto de Investigación Sanitaria, Universidad Complutense de Madrid, Madrid, Spain

**Keywords:** locally advanced rectal cancer, lateral pelvic lymph node, lateral pelvic lymph node dissection, East vs. West, surgical oncology

In the original article, there was a mistake in Figure 1 as published. **Figure 1** was our self-made drawing modified and adapted from:

Delaney CP. *Netter's Surgical Anatomy and Approaches*. Philadelphia, PA: Elsevier Saunders (2014).Radjindrin A, Shanmugam V. Does lateral pelvic lymph node matters in rectal cancer. *Glob Surg*. (2018) 4:3. doi: 10.15761/GOS.1000196

The mistake made was not to mention in the legend of Figure 1 and references that our self-made drawing was adapted from the sources mentioned previously. The authors apologize for any inconvenience, and under no circumstance was it the authors' intention to neglect to declare use of sources. The authors apologize for this error and state that this does not change the scientific conclusions of the article in any way. The original article has been updated.
